# Extracts from Discussions of the First Meeting of the Ohio State Dental Society

**Published:** 1867-06

**Authors:** 


### EXTRACTS FROM DISCUSSSIONS OF THE FIRST MEETING OF THE OHIO STATE DENTAL SOCIETY.

### DENTAL EDUCATION.

Dr. Taft—This subject of Dental education is an important field in the view which is presented—the relationship of teacher and pupil. In whatever aspect you view it, whether it is the education of the man in the office or in the school, or wherever you may place him with a view of educating him for this purpose—it is an important thing. It is important because of its own real value, because of the intrinsic value in the thing itself; and then again, it is important because there has been so much false practice in regard to it, because there has been in the profession so many mistakes.

It is resolved or regarded by most of our Associations, that in a short period a man cannot be prepared to practice all the departments of Dentistry, and hence they have passed resolutions like this; that we may not take a student for less than two years, some make it three years, others again pass resolutions that they will not take a student for a less pupilage than two years and the College instruction in addition. But then all over the country you will find men who will take young students; and for a compensation—for a poor, pitiful, miserable one hundred dollars—will take a young man in the office, keep him for a few months, and turn him out with a recommendation to the public—the recommendation that he is capable of practicing. This is all wrong, and it is doing injustice to the man himself. The preceptor, in the first place is doing himself injustice, is doing injustice to the man whom he thus takes, is doing injustice to his profession, and to the people at large. He is doing injustice to all parties; it is criminal so far as the people are concerned, and so far as the young man himself is concerned. Well, then what should be the standard? Can we fix any standard? It is true, that some young men will become better prepared in one year than others would in five, but then perhaps it is necessary to fix some limit with regard to the time, and the kind of study, and the things to be studied, in order to make it definite—in order to fix in the minds of all, that it is quite a task to make these attainments.

Now it is true, that in ten years many persons would not become prepared — after ten years thorough drilling, many persons would not be fit to practice Dentistry, in any of its departments, either in a manipulative or medical point of view. Others in a comparatively short period will be prepared, owing to their adaptation. Then, again, some will become capacitated in one particular—in the manipulative department—and be sadly deficient for a long time in other things, not being able to recognize and proceed for a long time in the treatment of various diseases; another will be able to diagnose with ease, while others again will be exceedingly bungling and clumsy in the command of their hands in manipulating and yet in a very little time, almost by a kind of intuition, will be able to look through and recognize diseased conditions.

All these things ought to be taken into account; and more than this, any one taking a young man for a student, ought to understand his capacity. He ought, in the first place, to understand his adaptability to this professsion, whether he has the natural disposition and inclination. Then, again, whether he has the elementary and early training proper to enable him to apply himself closely to study; this is a very important matter. And it is a matter of congratulation that in our profession, a movement is being made in this direction. A few years ago, ignorant men, comparatively sought to enter our profession. Now they are more and more highly educated, in literature and general science. Every year more and more, literary men seek to enter our profession. This is exceedingly encouraging. I remember years ago, that there were scarcely half a dozen students in a class of eighteen or twenty, who made any considerable pretensions to literary attainments. Now, by far, the greater proportion are what we would call good scholars. In one recent class of between forty and fifty, in the Ohio Dental College, I think over one-half were classical scholars; a far greater proportion than ever before. And you can scarcely imagine the difference between assisting or teaching such a class, and endeavoring to teach a class the greater proportion of whose members are illiterate. There is all the difference in the world, when you present an idea to a man who is able to understand and appreciate it, you see his countenance lighten up, it enthuses you, and enables him to go on and get facts that he otherwise could not; while if you have a class of illiterate men before you, and you give a most clear illustration — as clear as possible—it falls fruitless. I simply refer to this as a matter that is exceedingly gratifying.

Compare the discussions in the Associations now with those of a few years ago, and I think you will find a very great difference. This of course, I know, depends somewhat on the fact — that men of our profession have been associated together.

It then shows an improvement in this matter, and the members of the profession, who take students, are looking well to this thing and receiving men as students, whom they will be proud to introduce to their professional brethren, whom they will be proud to introduce in the future to other scientfic men.

This is a matter altogether in the hands of the members, profession, and they are responsible for the deficiencies, and the delinquencies that exist in the profession in this particular. It has been the custom heretofore that men would receive into their office, anybody who has one hundred dollars, and wish to stay two or three months, and work for them. Now, this is all wrong. The great and leading idea should be to make the right kind of a professional man of him. This much then in regard to the duty of the preceptor and the student. He should pursue such a course in his selection of students as will bring about the best results—as will secure in the ranks of the profession, a class of men of whom he will be proud hereafter as professional brethren and as scientific and literary men.

Now in reference to private instruction; many Dentists will say to the student: "Come into my office and I will teach you all you can get anywhere—and that in a short time; it is not necessary for you to know all these absurd things and studies in order to be a good Dentist; I can teach you everything that is necessary to make a good operator as well as anybody." That is not true. There is not a man living who can instruct the Dental student, as he should be taught in all the various branches pertaining to our profession. No man who will claim to be a first-class mechanical Dentist, and a first-class operative Dentist, and much less will you find, the man who is thoroughly competent to teach all these branches. And supposing he was competent to do so; how much time would it require? far more than any one could afford. Hence I regard it as the duty of the profession, to sustain the institutions we have, letting every student avail himself of all the means of proper professional instruction within his reach. I fully appreciate the advantages of private Dental pupilage. I think it an important thing if there is a good perceptor, one who will instill into the pupil proper principles; but there are a great many in the profession, and I am sorry to say it—who are not qualified for preceptors; and yet almost every one thinks himself well qualified to teach.

The institutions for instructing our young men, should be well sustained by the profession. They should be made just as good as possible. It is often said they are not what they ought to be. But why are they not? Because they are not receiving the countenance of all the men in the profession, who ought to sustain them. If the Colleges are not what they ought to be, the responsibility rests upon those who fail to sustain them; they have not been sustained by the profession as they ought to have been. If there are incompetent men in them, why are they there? Why do you suffer them to be there? Put them out and have better men, if there are any competent men in existence to teach. If there are incompetent men there, it is the fault of the profession. If the regulations and method of training is not what it should be, the fault rests on the profession.

But sustain these institutions, they have accomplished a work that cannot in any other wise be accomplished. Make them just as perfect as it is possible. As you sustain them so will their work be thoroughly accomplished; so will it result in advantage to all the profession. This and the next generation receive good from it—because, as the incoming members of the profession become enlightened, the light they get and the influence they have, is thrown out and exerted on every member of the profession.

Then sustain your institutions. The advantages are manifested in many respects. See what has been accomplished through them. Cut off from the profession every good influence that has ever gone out from the Dental Colleges of the United States, and where is your profession? What is it worth? I do not say there are not good men who have not been in the Colleges; take away all the influences that has ever emanated from the Schools and Colleges, and where is your professional literature? The great majority of the prominent men of the profession in the country, have grown up, in a great degree, under the influence of these institutions, and I do not believe there is a man anywhere who may claim to have grown up wholly independent of these things.

It is then the duty of every member of the profession, to sustain our Colleges, and if they are not what they ought to be, make them better; I know there are many slurs thrown out against the Colleges. But Why? Is it against an institution, that a thick-headed student comes in occasionally, and never amounts to anything? Is it anything against an institution, that a man goes into it, and afterwards becomes a scalliwag, and he remains so ever afterwards? It is nothing against the institution. It is nothing against an institution to have received as an honest man, one who afterwards proved to be a quack. And it is nothing against our institutions that there are just as good men outside of them, as were ever in them. They have been valuable; they might have been eminently more so, had they been properly sustained.

I am not making a special plea for the Colleges, but for the profession and for humanity.

It has been said, that degrees are some times conferred on young men who ought not to have them. That perhaps, is the case; it is the case in every College in the land; it is so in every literary institution, and I think we have, perhaps, as thorough protection in this matter as it is possible to make. I will simply refer to the method of examination that is adopted in one of the Dental Colleges at least. At the close of the last session, each teacher wrote his list of questions on the black-board, and the members of the class sat in their seats and wrote the answers to those questions. These questions ranged in numbers from twelve to forty. Each student remained in his seat till the answers were written, and passed in, seventy-five per cent. of which were required to be correct.

Dr. Watt—In addition to that it was left for the members of the Faculty to say whether the answers to his questions were correct or not. He might want to make them believe that he taught so well that his questions were always answered right; the answers of the students were read to the Faculty and they voted on all the answers. So, if the teacher had a pet student there was no chance for him, as the teacher was not left to decide.

Dr. Keely—I know at this time it is looked upon as of far more importance than formerly—this matter of educating students. Who is he, I want to know, in the first place? I want to know if he is an intelligent, honest young man, if he is a young man that I would be proud to introduce to my family and female friends. This I consider very important, and further than that I always felt that I never would wish a young man to go out of my office and state to the public that he was a student of mine unless he was qualified—at least as much so as it was possible for me to make him as a student for two years. Farther than this, I have always had my students pledge their honor that they would, as soon as possible, after they got away, take a regular course in some Dental College, and many have done it; several have not finished their course; several have taken one course and I know they will finish that course. I know that I do not do much for the Ohio Dental College, or for any particular Dental College; I do not wish you to understand, gentlemen, that I pledge my students to attend the Ohio Dental College, but some Dental College. Here is a matter that I think the most of us are at fault in not, when taking young men into the profession, urging them to take a course at the Dental College. Young men who have been in practice a few years by studying a little at a Dental College, have qualified themselves in due time, while some it did not require so long a time.. This is the point I want to get at to urge upon every member of this society the importance of every young man attending some Dental College.

Dr. James—This last resolution is very good but it seems to me that there is a large portion of intelligent, worthy young men who will find it, so far as their finances are concerned, an utter impossibility to attend a Dental College. Would it not be well to insert in that resolution, "provided their finances will allow it, to take two courses of lectures in a College.

Dr. Cady—I would like to know how much time is required at the Dental College at Cincinnati, and about what the expense is including board. I ask for this reason:—We are applied to now by two intelligent young men, one of them is a College graduate of this city, twenty-four years of age. He has money, as far as that is concerned, but I want to know what the terms are.

And another thing I enquire, if a young man comes in my office and remains two years and then enters the Dental College whether it helps him towards getting through; hurries him through; or can he go there from the farm and get through just as quick as if he had his two years instruction in my office? I would like to understand these things a little clearer.

Dr. Watt—The terms are published in the advertisements and announcements. I believe it is said a satisfactory Dental pupilage and two courses of lectures are required for graduation.

Dr. Taft—That depends altogether on the character of the pupilage. As I said awhile ago, better not have any Dental instruction at all than to have it of an improper kind. If a young man is encouraged and aided on in studying the elementary principles, anatomy, pathology, physiology, etc., he can get on and follow a lecturer in his course much better; but if a young man comes perfectly green, he will find it exceeding hard work to follow up the course of instruction that is given him from the different chairs. The same thing is true with regard to the practical departments. If he is properly trained by a preceptor, the more of it he has the better, and the faster he can go on picking up ideas and the more perfect he will become in the end. But sometimes young men have been taken in with very little preparation—very little indeed—they will receive them if they have had but a few months pupilage or training, but then they have to pursue their studies closely all the while.

You will understand this, that every young man has to pass an examination before he can receive a diploma. He must come up to a certain standard if it takes him ten years to do it, but if he does it in two sessions and a short pupilage all well. If he comes up and stands a thorough examination though he may have made all his attainments in the College, that is sufficient. He has to present a piece of mechanical work for the inspection of the Faculty, and the teacher of operative Dentistry reports upon the character of his operations to the Faculty. The question is asked what is the character of his operations? Does he put in good fillings? It is all brought out, and generally, the members of the Faculty see these operations, they come in uniformly and see them.

It is not only a mere oral or written examination that decides the matter, but in operative ability he must come up to the standard or he cannot pass; and the more good Dental pupilage he can have, the better. Of course, any one taking a student for a two years pupilage, it is ordinarily regarded that he may take his two courses of lectures when he chooses, each occupying four months, eighteen weeks of instruction, aside from the holidays; after he takes this course of instruction for two years, if he stands an examination he receives his diploma. But it is the fewest number indeed that can pass an examination in two years

The expenses the first year in the College is one hundred and ten dollars, and it may be, the same amount for board, making for contingent expenses about two hundred and fifty dollars for the first, and three hundred for the second.

Dr. Cady—What difference does it make about a student remaining in our office, you say you do not give him a sheepskin till he has arrived at a certain standard.

Dr. Taft—I think it would be well that he take two years pupilage first, and two years in the College afterwards.

Dr. DeCamp—I will state what has been our custom for a number of years and the brethren here may determine whether we are right or wrong. As Dr. Taft remarked, we make it a rule, and have kept it up, that we would take no young man as a student unless, in the first place, he had a good English education, and was a moral young man, upright in every respect, and a young man with proper capacity to make a man with proper training. We adhere to these terms. We have recently taken two on these terms. We bind them to stay two years and take a regular course in some Dental College. The course of instruction in the office, as a matter of course, is a thorough course of reading in the text-books.

